# Comparison of Mid and Near-Infrared Spectroscopy to Predict Creatinine, Urea and Albumin in Serum Samples as Biomarkers of Renal Function

**DOI:** 10.3390/bios15120786

**Published:** 2025-12-01

**Authors:** Diogo Serrano, Paulo Zoio, Luís P. Fonseca, Cecília R. C. Calado

**Affiliations:** 1 iBB—Institute for Bioengineering and Biosciences, i4HB—The Associate Laboratory Institute for Health and Bioeconomy, IST—Instituto Superior Técnico, Universidade de Lisboa, Av. Rovisco Pais1, 1049-001 Lisbon, Portugal; diogosserrano@gmail.com (D.S.);; 2 ISEL—Instituto Superior de Engenharia de Lisboa, Instituto Politécnico de Lisboa, R. Conselheiro Emideo Navarro 1, 1959-007 Lisbon, Portugal

**Keywords:** kidney diseases, IR spectroscopy, biomarkers, monitoring

## Abstract

It is relevant to develop new technologies to enable the intensive monitoring of kidney function in a minimally invasive, rapid, and economic way. With this goal, the current work compared infrared spectra in the mid- (MIR) and near-infrared (NIR) regions to predict the biomarkers of kidney function, i.e., serum creatinine, urea, and albumin. After the evaluation of diverse spectra pre-processing methods and spectral regions, it was possible to develop, in either spectral region, good to excellent regression models to predict these three biomarkers, with determination coefficients (R^2^) of over 0.9 and relatively low root mean squared error (RMSE). The two techniques are complementary, since the MIR spectroscopic platform presents the advantage of enabling high-throughput analysis, as it includes the sample analysis based on a micro-plate with 96 wells, only requiring a simple dehydration step before spectra acquisition, while the NIR spectroscopic platform enables the direct analysis of the serum solutions in real time. Therefore, both platforms present complementary characteristics with a high potential to enable reagent-free and frequent monitoring of kidney function and incorporation into point-of-care diagnostic systems.

## 1. Introduction

Chronic kidney disease (CKD) represents a significant global health concern, affecting approximately 10% of the worldwide adult population [1]. Furthermore, the prevalence of CKD has been on the rise, with studies indicating a substantial increase in cases over recent decades, underscoring the need for effective prevention and management strategies. CKD is characterized by a gradual loss of kidney function over time, that if left unmanaged, often progresses to end-stage renal disease (ESRD). Major risk factors contributing to CKD include diabetes mellitus, hypertension, and glomerulonephritis [2]. Acute kidney injury (AKI), a sudden decline in kidney function, is intricately linked with CKD, acting as both a precursor and accelerant, while pre-existing CKD increases AKI risk [3]. This bidirectional relationship emphasizes the importance of early detection and intervention in both conditions to prevent progression and associated complications.

The impact of CKD and AKI extends beyond individual health, imposing substantial financial, social, and environmental burdens. Economically, the management of kidney disease and its complications accounts for a significant portion of healthcare expenditure, particularly in advanced stages requiring dialysis or transplantation. Socially, CKD can lead to reduced quality of life, loss of productivity, and increased dependency. Environmentally, dialysis treatments contribute to considerable water and energy consumption, as well as medical waste generation, highlighting the need for sustainable healthcare practices [4,5,6].

Early diagnosis of CKD and AKI, and the monitoring of the disease, is crucial in mitigating disease progression and associated complications. Traditional diagnosis and staging of kidney disease rely primarily on glomerular filtration rate (GFR) assessment, typically calculated from serum creatinine and often complemented by serum urea [7]. Despite the availability of these screening methods, CKD often remains undiagnosed until its advanced stages, underscoring the need for improved diagnostic tools that may be applied in frequent monitoring. To promote the monitoring of kidney disease, it is therefore urgent to develop point-of-care methods enabling the prediction of the organ function, that could be conducted in a cheap and fast mode. With that goal, a diverse range of researchers have explored infrared (IR) spectroscopy [8], since it is a reagent-free analysis, is economic, and provides immediate results, and consequently can be useful for intensive monitoring and in point-of-care testing applications, such as at the bedside in care units or intensive care units.

Infrared spectroscopy encompasses both the mid-infrared (MIR) region, usually ranging between 400 and 4000 cm^−1^, and the near-infrared (NIR) region, usually ranging between 4000 and 12,000 cm^−1^. The advantage of MIR spectroscopy is that it reflects fundamental molecular vibrations and consequently gives rise to stronger and better-defined absorbance bands when compared to NIR, which reflects overtones and combinations of molecular vibrations [9,10]. Furthermore, MIR spectra of complex biological samples, such as blood, are sensitive to a higher diversity of functional groups, such as C-C, C=C, C-O, C-N, C-H, O-O, O-P, N-H, and O-H bonds, while NIR covers most groups containing the hydrogen atom, such as C-H, N-H, O-H, and S-H bonds [11]. Consequently, MIR spectra are theoretically more informative about the sample’s biomolecular composition. However, MIR is highly sensitive to water, often requiring sample dehydration for transmission detection or more complex methods of detection [12], while NIR spectroscopy may be applicable to in situ analysis, and with cheaper portable equipment. Nonetheless, comprehensive comparative assessments between MIR and NIR spectroscopy using complex biological matrices such as serum remain limited, particularly regarding how pre-processing strategies influence model robustness and predictive accuracy.

Due to the potential of MIR and NIR spectroscopy to monitor renal function, a diverse range of authors have been exploring these techniques to monitor diseases such as CKD and AKI. However, most of the works associated with NIR spectroscopy focused on evaluating oxygenation levels [13,14], and the MIR-associated techniques mostly focused on detecting urea [15], or detecting end-stage kidney disease [16,17]. Another example of the application of these techniques is plasma analysis to discriminate between AKI and healthy controls [18]. Despite the merits of these previous works, a comparison of MIR and NIR spectroscopy is missing, to predict the simultaneous concentration of creatinine, urea, and albumin. The current study directly addresses this gap with particular attention given to evaluating the impact of different spectral pre-processing approaches and spectral sub-regions to enhance model performance.

In the current work, the MIR spectra of serum samples were acquired using a micro-plate with 96 wells to conduct a high-throughput analysis, requiring a simple dehydration step before spectra acquisition, while NIR spectroscopy was conducted by in situ and in real time analysis using a fiber-optic probe. The serum NIR and MIR spectra were used to develop regression models to determine the creatinine, urea, and albumin concentration.

## 2. Materials and Methods

### 2.1. Biological Samples Preparation

A diverse range of solutions were prepared in fetal bovine serum (Biowest, Nuaillé, France) at a final dilution of 1/5 (*v*/*v*) in water. The following 60 solutions were prepared. Creatinine (Merck, Darmstadt, Germany) (mg/dL): 0.56; 0.63; 0.7; 0.84; 0.98; 1.12; 1.19; 1.36; 1.76; 2.16; 2.56; 2.96; 3.56; 4.56; 5.56; 6.56; 7.56; 8.16; 8.96; 9.76. Urea (Merck, Germany) (mg/dL): 6.4; 12.4; 18.4; 24.4; 30.4; 36.4; 42.4; 48.4; 54.4; 60.4; 72.4; 84.4; 96.4; 108.4; 120.4; 132.4; 144.4; 162.4; 180.4; 198.4. Albumin (Bovine Serum Albumin, lyophilized powder, Sigma-Aldrich, St. Louis, MO, USA) (g/dL): 0.29; 0.49; 0.69; 0.82; 1.29; 1.62; 1.96; 2.29; 2.62; 3.62; 3.96; 4.29; 4.62; 4.96; 5.29; 5.62; 6.29; 6.96.

### 2.2. MIR Spectroscopy

20 µL of each solution was placed on IR-transparent Si microtiter plates with 96 wells (Bruker Optics, Ettlingen, Germany) and subsequently dehydrated for 2.5 h in a vacuum desiccator setup (ME2, Vacuubrand, Wertheim, Germany). The MIR spectra were recorded in transmission mode with an HTS-XT associated with a Vertex-70 spectrometer (Bruker Optics), using a spectral resolution of 4 cm^−1^ and 40 scans per sample in the spectral range between 4000 and 400 cm^−1^. Each solution was analyzed in triplicate with a background spectrum obtained from an empty well.

### 2.3. NIR Spectroscopy

Spectra were acquired using an NIR transflection fiber optic probe, IN-271P (Bruker Optics), with a path length of 2 mm, coupled to a Vertex-70 spectrometer (Bruker Optics) by submerging it in 2 mL of each solution. A reference atmospheric air spectrum was acquired before the probe was inserted into the solutions. Spectra were collected in the 12,500–4000 cm^−1^ range, consisting of 32 co-added scans with 8 cm^−1^ resolution. The scanner velocity was set to 10 kHz, and the aperture setting was defined as 6 mm. Background signal removal (blank) of the atmosphere was performed before every sample read.

### 2.4. Spectra Pre-Processing and Processing

Spectra atmospheric compensation was applied to all MIR spectra. Combinations of the following spectral pre-processing methods were evaluated: baseline correction using the Rubber Band method (BC), Standard Normal Variate (SNV), Unit Vector Normalization (UVN), and first (1D) and second (2D) derivatives using a Savitzky–Golay filter with a second-order polynomial and 15 smoothing points.

Partial Least Squares (PLS) regression models were elaborated with a maximum of 10 latent variables and mean-centered data using the KERNEL PLS algorithm. A total of 60 spectra were considered for each target molecule, since triplicate spectra were acquired for each of the 20 solutions. A cross-validation method was applied, in which 5 cycles of validation were conducted, where for each cycle, 80% of randomly selected samples were used for training (i.e., 48), and the remaining (i.e., 12) for validation. The models’ performance was evaluated considering the coefficient of determination (R^2^), the root-mean-square error (RMSE), and the number of latent variables. To obtain better regression models, defined sub-regions of the NIR spectrum were evaluated, e.g., the regions associated with the third overtone (12,500–8400 cm^−1^), the second overtone (9100–6250 cm^−1^), and the first overtone (6700–5000 cm^−1^), and the combination bands region (5200–4000 cm^−1^).

Spectra atmospheric compensation and baseline correction were conducted with OPUS^®^ software, version 6.5 (Bruker, Germany), while remaining spectra pre-processing and processing methods were conducted with Unscrambler^®^ X 10.4 software (CAMO software AD, Oslo, Norway).

## 3. Results and Discussion

### 3.1. Preparation of Serum Samples Simulating Kidney Disease

A total of 20 solutions of creatinine in serum were prepared, with final concentrations between 0.56 and 9.76 mg/dL, to simulate the creatinine concentration in blood from healthy individuals to patients with advanced CKD. In clinical practice, normal serum creatinine levels typically range from 0.7 to 1.2 mg/dL in males and 0.5 to 1.0 mg/dL in females, while values exceeding 2.0 mg/dL are commonly associated with moderate to severe CKD [19]. Additionally, serum solutions with urea concentrations ranging between 6.4 and 198.4 mg/dL were also considered, since blood urea nitrogen (BUN) is another critical biomarker routinely used to evaluate renal function. Normal blood urea levels typically range from 7 to 20 mg/dL in healthy adults [20]. Elevated urea concentrations, particularly levels exceeding 50 mg/dL, are commonly associated with impaired renal function and are indicative of uraemia in advanced stages of CKD [21]. The upper end of the concentration range used in this study (nearly 200 mg/dL) was chosen to simulate severe renal impairment, including end-stage renal disease (ESRD), where urea levels can rise dramatically due to reduced glomerular filtration and accumulation of nitrogenous waste products in the blood [21]. While serum creatinine remains the primary analyte for estimating eGFR, BUN provides complementary information, particularly in acute settings or when evaluating the urea-to-creatinine ratio. This ratio can help differentiate between prerenal and intrinsic causes of kidney dysfunction. Moreover, BUN is often included in diagnostic panels such as the blood urea nitrogen-to-creatinine ratio (BUN:Cr), which can offer insights into hydration status, protein metabolism, and catabolic states [21].

Solutions of serum with albumin concentrations between 0.29 and 6.96 g/dL were also considered, due to the relevance of albumin in assessing nutritional status and CKD stage. Indeed, although albumin is more commonly assessed in urine (as albuminuria) to detect early kidney damage, serum albumin remains a valuable biomarker for evaluating the systemic effects of CKD and guiding nutritional and therapeutic interventions [21].

The concentration range encompassed hypoalbuminemia to normoalbuminemia states, reflecting the clinical variability observed in CKD patients. In healthy individuals, normal serum albumin levels typically range from 3.5 to 5.0 g/dL. Hypoalbuminemia—defined as serum albumin levels below 3.5 g/dL—is frequently observed in patients with advanced CKD and is associated with increased morbidity and mortality [20]. Low albumin levels may result from protein-energy wasting, systemic inflammation, or proteinuria, all of which are common in progressive kidney disease [21]. Conversely, elevated serum albumin levels are rare and may reflect dehydration or laboratory artifacts.

### 3.2. MIR Spectroscopic Analysis

**Figure 1:**
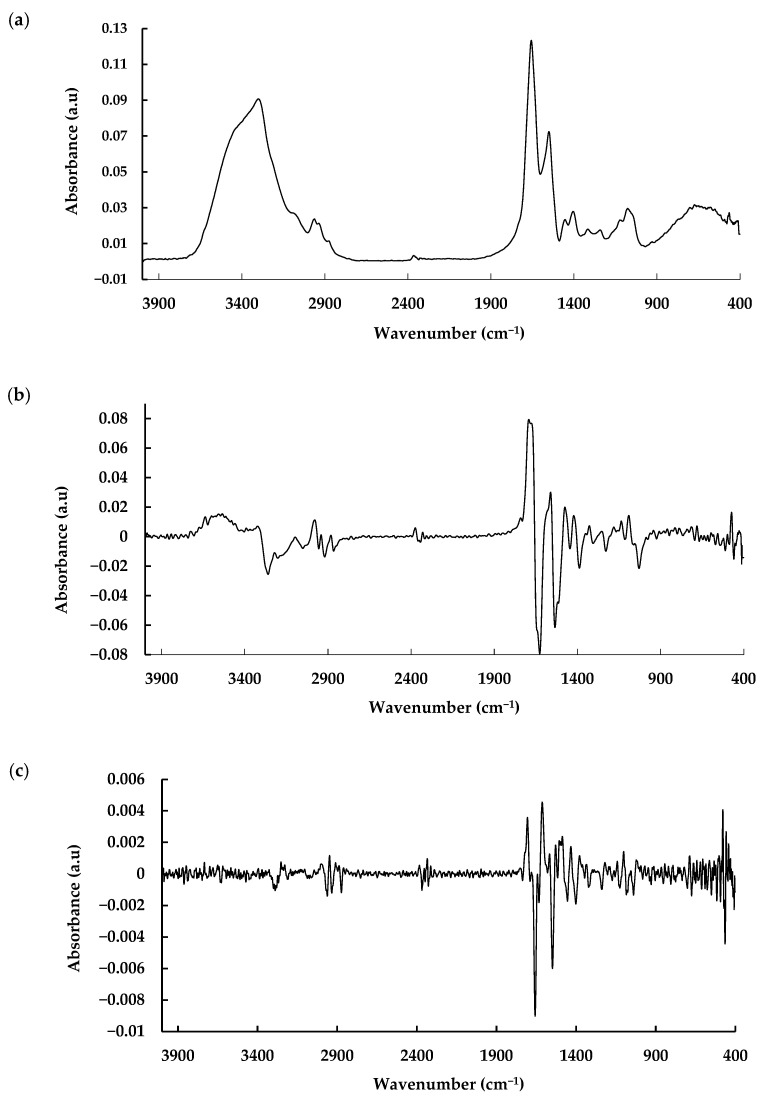
MIR spectra of a serum solution, with (**a**) baseline correction; (**b**) first derivative; and (**c**) second derivative spectra.

The MIR spectra of the serum solutions were acquired in transmission mode after a dehydration step and using a plate with 96 micro-wells. Diverse PLS regression models were built to predict the concentration of creatinine, urea, and albumin, based on spectra pre-processed with baseline correction (BC), or first (1D) or second (2D) derivatives with and without normalization SNV (Figure 1, Table 1). The models’ performance was evaluated by the R^2^, the RMSE, and the number of latent variables for both calibration and validation datasets. As expected, the models’ performance on the calibration dataset was better than on the validation dataset (Table 1).

**Figure 2:**
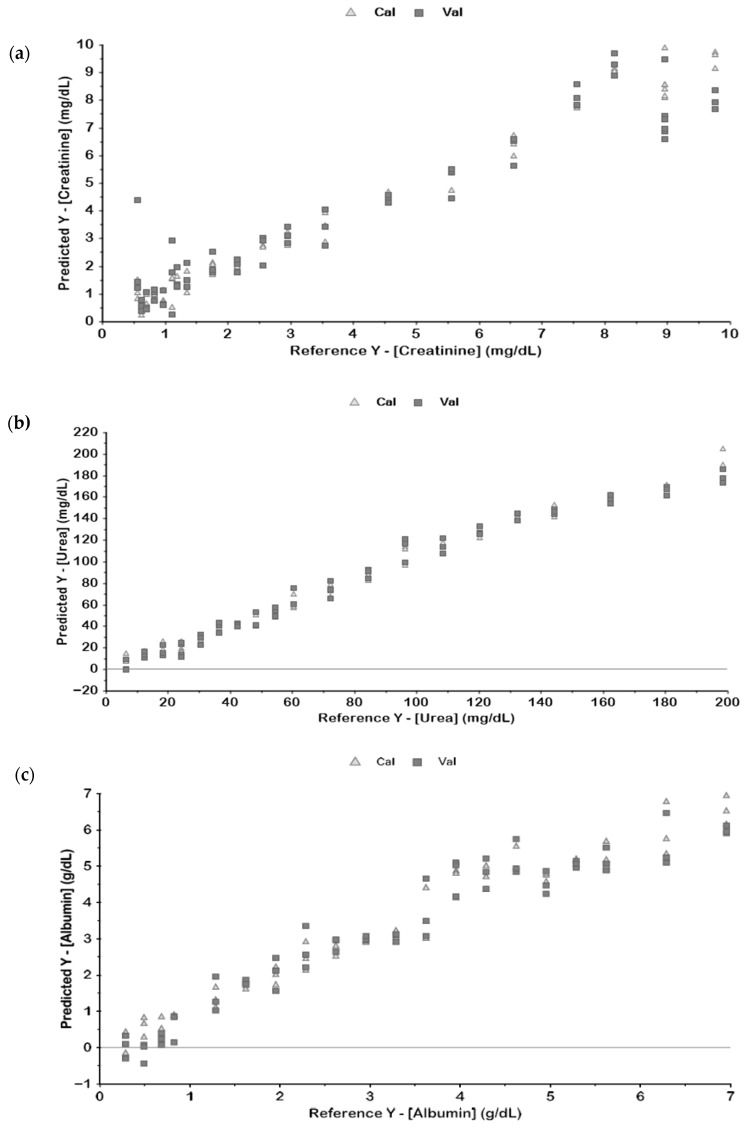
Reference (real) vs. predicted concentrations for serum creatinine (**a**), urea (**b**), and albumin (**c**) of the PLS regression models based on the baseline corrected, normalized, and first derivative pre-processed serum MIR spectra (BC + SNV + 1D—Figure 1).

In general, it was possible to develop good PLS models based on MIR spectra to predict creatinine, urea, and albumin, with R^2^ > 0.90 and low RMSE for the validation dataset. Interestingly, the best models were based on normalized spectra, with or without derivatives. As an example, Figure 2 represents the regression models for these three variables based on the normalized first derivative spectra, indicating a validation dataset R^2^ > 0.90 and RMSE lower than 10% for the three variables.

The excellent models to predict these three analytes in plasma are in accordance with those obtained by other authors. For example, Low-Ying et al. [22] predicted the urea concentration in blood for a similar range of concentrations (15–120 mg/dL), with regression models with a slightly worse R^2^ (of 0.94 versus 0.98 for our best model). Jessen et al. [23] developed regression models to predict urea and albumin in plasma, with R^2^ values of 0.99 and 0.92, respectively, like our best models with 0.98 and 0.92, respectively. Henn et al. [24] predicted urea and creatinine concentrations in dialysates, with regression models with an R^2^ and a RMSE for urea of 0.99 and 6.6 mg/dL, respectively, and for creatinine with an R^2^ of 0.98 and a RMSE of 1.5 mg/dL, showing similar performance to our best models (Table 1). Lastly, in blood, Hoşafçı et al. [25] predicted urea concentration with an R^2^ of 0.98 and a RMSE of 4.4 mg/dL, and albumin concentration with an R^2^ of 0.97 and a RMSE of 0.15 g/dL.

One advantage of the acquisition mode applied in the current work was the high-throughput analysis implemented, based on micro-plate analysis, enabling the automatic acquisition of 96 samples, simplifying the large-scale analysis seen in clinical settings, in contrast to the analysis conducted by all previous works. Furthermore, a very small amount of serum was needed for analysis (of 20 µL), obtained, for example, from a simple finger prick, i.e., from a minimally invasive method.

### 3.3. NIR Spectroscopic Analysis

**Figure 3:**
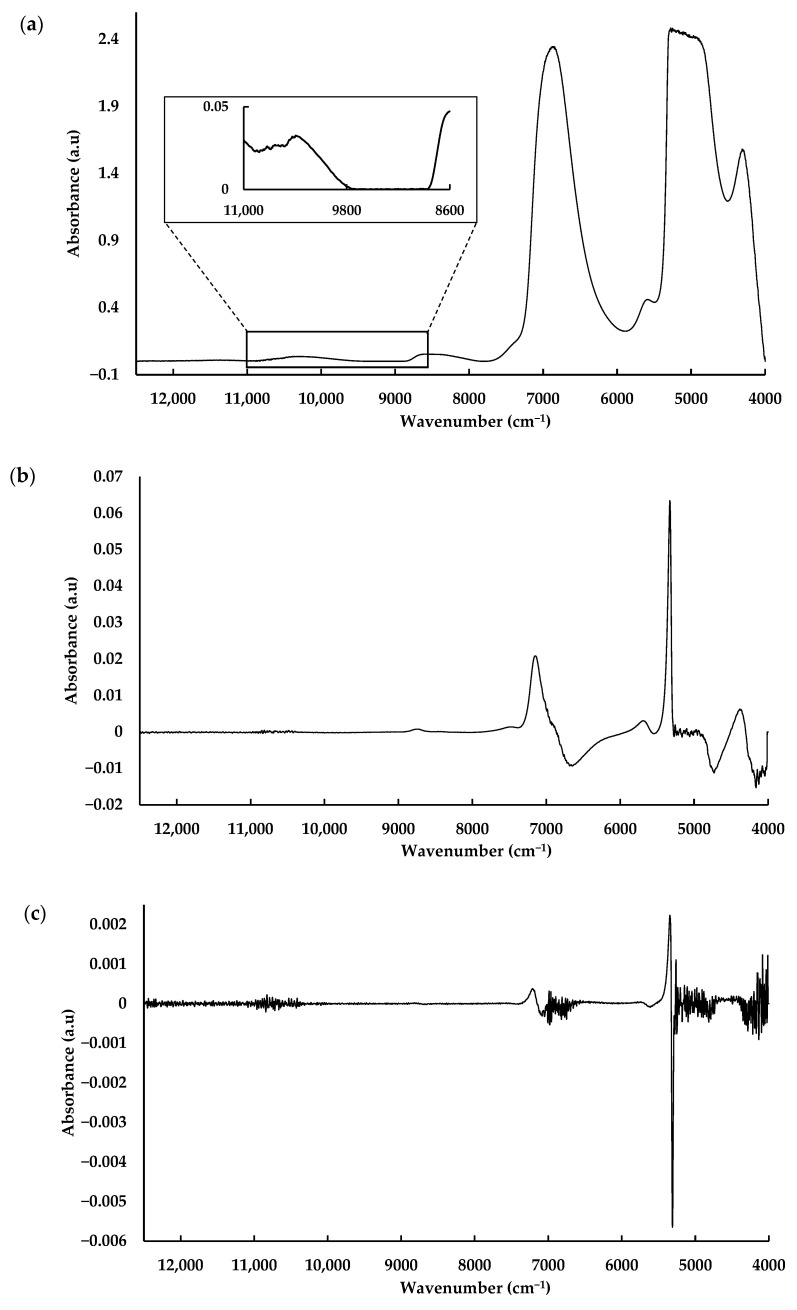
NIR spectra of a serum solution, with (**a**) baseline correction with the 11,000–8600 cm^−1^ region highlighted, (**b**) first derivative, (**c**) and second derivative spectra.

NIR spectra were acquired directly from serum solutions using a fiber optic transflectance probe, enabling real-time analysis, i.e., without the need for the dehydration step conducted previous to MIR spectra acquisition. After similar pre-processing methods (Figure 3), like those applied with MIR spectra, it was possible to develop a good model to predict urea, with an R^2^ for the validation dataset of 0.79, and an excellent model to predict albumin, with an R^2^ for the validation dataset of 0.99 (Table 2). The model to predict albumin was even slightly better than the one based on MIR spectra (Table 1 and Table 2). It was not possible to develop an accurate model to predict creatinine concentration based on the whole NIR spectra.

**Figure 4:**
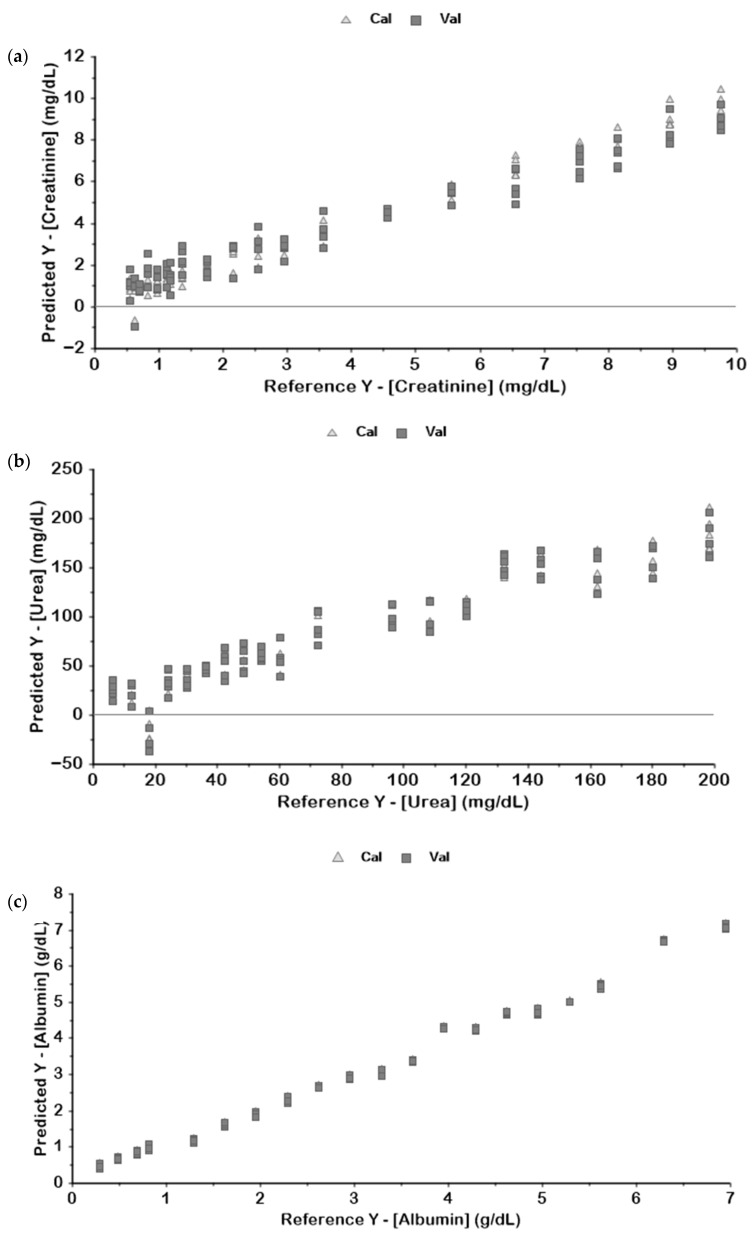
Predicted vs. Reference curves of the best PLS regression models obtained from NIR spectra for creatinine (**a**), urea (**b**) and albumin (**c**).

To improve the model’s performance, several spectral sub-regions were tested instead of using the whole spectrum, to diminish the noise of segments of the spectrum that had no correlation with metabolite concentration. These models were built with spectra after baseline correction and normalization (Table 3). Indeed, it was possible to develop excellent models to predict creatinine based on the region between 8600 and 11,000 cm^−1^, with a slightly higher R^2^ of 0.94 vs. 0.91, and a lower RMSE of 0.73 vs. 0.99 mg/dL, comparatively with the ones obtained with the whole MIR spectra, respectively (Figure 4, Table 1 and Table 3). The best model to predict urea was built with the NIR region between 4400 and 4700 cm^−1^, which led to a model slightly worse than the one obtained with the MIR spectra, with an R^2^ of 0.90 vs. 0.98, and a RMSE of 19.0 vs. 9.0 mg/dL, respectively (Figure 4, Table 1 and Table 3). The best model developed to predict urea based on NIR spectra presenting a similar performance was the one developed by Henn et al. [24] for hemodialysis liquid, with an R^2^ of 0.99.

## 4. Conclusions

In the present work, it was possible to develop excellent predictive models for biomarkers of kidney function, i.e., the concentration of creatinine, urea, and albumin in serum, with an excellent determination coefficient and lower RMSE, based on either MIR spectroscopy or NIR spectroscopy. These models were valid for a wide range of concentrations, covering healthy status to the most severe CKD stage. An advantage of the present work, in relation to other authors, is that most works using NIR and MIR were not conducted in serum samples, but with less complex samples such as aqueous systems [26], urine [27,28,29], saliva [30], and dialysate [24]. The few studies based on blood analysis, or its corresponding serum or plasma [31,32,33], did not predict the three analytes in tandem, as in the current work, i.e., creatinine, urea, and albumin. Furthermore, they did not conduct a direct comparison of the MIR and the NIR platform.

In the current work, all models presented very high performance, as in addition to covering a wide range of physiological concentrations, they also presented, for the validation dataset, correlation coefficients equal to, or higher than, 0.90 and low errors, both in MIR and NIR. As expected, the MIR spectroscopy-based platform presented an excellent model to predict urea concentration (R^2^ = 0.98 and RMSE = 9.1 mg/dL), compared to the models obtained with the NIR-based platform (with R^2^ = 0.90 and RMSE = 19.0 mg/dL). However, the NIR-based platform after optimization of the spectral region delivered the best models to predict the creatinine and albumin concentrations, obtaining for the validation dataset correlation coefficients equal to, or higher than, 0.94 and RMSE values of 0.73 mg/dL and 0.19 g/L, respectively. The MIR spectroscopic platform presents the advantage of enabling high-throughput analysis, since it includes a micro-plate with 96 wells, only requiring a simple dehydration step before spectra acquisition, while the NIR spectroscopic platform enables the direct analysis of the serum solutions in real time. Therefore, both platforms, i.e., MIR- and NIR-based systems, present complementary characteristics that can be very useful for monitoring and controlling kidney diseases.

## Figures and Tables

**Table 1:** PLS regression models to predict the serum creatinine, urea, and albumin from MIR spectra. Calibration and validation sets’ performance was evaluated by the R^2^ and root mean square error values, the latter having units of mg/dL for creatinine and urea and g/dL for albumin. The best models are highlighted in bold.

Metabolite	Pre-Processing	Latent Variables	Calibration		Validation	
			R^2^	RMSE	R^2^	RMSE
Creatinine	Raw	8	0.97	0.53	0.75	1.54
	BC	9	0.98	0.41	0.78	1.50
	BC + SNV	10	0.98	0.43	0.91	0.99
	**BC + SNV + 1D**	**5**	**0.98**	**0.43**	**0.90**	**0.99**
	BC + SNV + 2D	6	0.98	0.38	0.84	1.31
Urea	Raw	4	0.98	8.6	0.96	11.7
	BC	3	0.98	8.4	0.97	10.3
	BC + SNV	2	0.96	10.8	0.96	11.6
	**BC + SNV + 1D**	**3**	**0.99**	**6.6**	**0.98**	**9.1**
	BC + SNV + 2D	2	0.96	11.8	0.94	14.0
Albumin	Raw	4	0.95	0.43	0.93	0.53
	BC	8	0.99	0.21	0.95	0.45
	BC + SNV	7	0.96	0.39	0.92	0.54
	**BC + SNV + 1D**	**4**	**0.96**	**0.39**	**0.92**	**0.57**
	BC + SNV + 2D	3	0.94	0.49	0.82	0.84

**Table 2:** PLS models built with the whole NIR spectra of serum solutions to predict creatinine, urea, and albumin using several spectral pre-processing methods. Calibration and validation sets’ performance is evaluated by the R^2^ and root mean square error values, the latter having units of mg/dL for creatinine and urea and g/dL for albumin.

Metabolite	Pre-Processing	Latent Variables	Calibration		Validation	
			R^2^	RMSE	R^2^	RMSE
Creatinine	Raw	10	0.97	0.54	0.25	2.63
	BC	8	0.89	0.98	0.21	2.70
	BC + SNV	9	0.96	0.59	0.28	2.58
	BC + SNV + 1D	2	0.09	2.87	0.07	2.92
	BC + SNV + 2D	---	---	---	---	---
	BC + UVN	10	0.98	0.39	0.29	2.58
	1D	2	0.09	2.88	0.06	2.96
	2D	2	0.11	2.84	0.06	2.95
Urea	Raw	5	0.94	14.63	0.79	27.21
	BC	5	0.93	15.10	0.69	32.77
	BC + SNV	5	0.95	13.14	0.7	32.38
	BC + SNV + 1D	1	0.64	35.14	0.23	52.20
	BC + SNV + 2D	1	0.55	39.29	0	60.41
	BC + UVN	5	0.96	12.32	0.71	31.86
	1D	1	0.65	34.63	0.23	51.8
	2D	1	0.55	39.25	0.02	58.60
Albumin	Raw	2	0.99	0.22	0.99	0.23
	BC	3	0.99	0.17	0.99	0.18
	BC + SNV	3	0.98	0.23	0.98	0.30
	BC + SNV + 1D	3	0.98	0.26	0.98	0.29
	BC + SNV + 2D	6	0.98	0.24	0.8	0.89
	BC + UVN	4	0.99	0.20	0.99	0.23
	1D	3	0.98	0.25	0.98	0.27
	2D	7	0.99	0.22	0.80	0.88

**Table 3:** PLS models built with sub-regions of the NIR spectra to predict the serum creatinine, urea, and albumin, pre-processed with baseline correction and unit vector normalization. The best models are highlighted in bold.

Metabolite	Spectral Sub-Region	Latent Variables	Calibration		Validation	
			R^2^	RMSE	R^2^	RMSE
Creatinine	Full Spectrum	10	0.98	0.390	0.29	2.579
	3rd Overtone	7	0.98	0.377	0.67	1.745
	2nd Overtone	1	0.04	2.944	0.02	3.010
	1st Overtone	1	0.04	2.944	0	3.044
	Combination Bands	3	0.16	2.768	0.10	2.890
	**11,000–8600**	**6**	**0.98**	**0.399**	**0.94**	**0.727**
	7800–7050	1	0.01	2.989	0	3.140
	5800–5300	1	0.04	2.955	0.01	3.030
	4700–4400	1	0.02	2.977	0	3.032
Urea	Full Spectrum	5	0.96	12.324	0.71	31.856
	3rd Overtone	5	0.99	6.769	0.73	30.809
	2nd Overtone	3	0.51	40.803	0.41	45.911
	1st Overtone	3	0.68	33.050	0.48	43.191
	Combination Bands	4	0.9	18.787	0.66	34.740
	11,000–8600	4	0.94	13.927	0.75	29.719
	7800–7050	3	0.54	39.824	0.44	44.019
	5800–5300	10	0.82	25.044	0.58	38.200
	**4700–4400**	**4**	**0.92**	**16.359**	**0.90**	**19.038**
Albumin	Full Spectrum	4	0.99	0.200	0.99	0.229
	3rd Overtone	5	0.99	0.212	0.95	0.429
	2nd Overtone	3	0.99	0.231	0.98	0.241
	1st Overtone	2	0.98	0.266	0.98	0.276
	Combination Bands	4	0.98	0.247	0.98	0.290
	11,000–8600	6	0.99	0.192	0.97	0.334
	7800–7050	5	0.98	0.284	0.97	0.355
	5800–5300	5	0.99	0.184	0.99	0.211
	**4700–4400**	**2**	**0.99**	**0.187**	**0.99**	**0.191**

## Data Availability

The data presented in this study will be available on request from the corresponding author.
